# Particle Radiotherapy for Skull Base Chondrosarcoma: A Clinical Series from Italian National Center for Oncological Hadrontherapy

**DOI:** 10.3390/cancers13174423

**Published:** 2021-09-02

**Authors:** Giulia Riva, Iacopo Cavallo, Sara Gandini, Rossana Ingargiola, Mattia Pecorilla, Sara Imparato, Eleonora Rossi, Alfredo Mirandola, Mario Ciocca, Ester Orlandi, Alberto Iannalfi

**Affiliations:** 1 Clinical Department, National Center for Oncological Hadrontherapy (CNAO), 27100 Pavia, Italy; iacopo.cavallo@cnao.it (I.C.); rossana.ingargiola@cnao.it (R.I.); mattia.pecorilla@cnao.it (M.P.); Sara.imparato@cnao.it (S.I.); eleonora.rossi@cnao.it (E.R.); alfredo.mirandola@cnao.it (A.M.); mario.ciocca@cnao.it (M.C.); ester.orlandi@cnao.it (E.O.); alberto.iannalfi@cnao.it (A.I.); 2 Department of Oncology and Hemato-Oncology, University of Milan, 20122 Milan, Italy; 3 Department of Experimental Oncology, European Institute of Oncology, IRCCS, 20139 Milan, Italy; sara.gandini@ieo.it

**Keywords:** proton therapy, particle therapy, carbon ion therapy, multimodal treatment, toxicity

## Abstract

**Simple Summary:**

Skull-base chondrosarcoma is a rare locally aggressive or malignant group of cartilaginous neoplasm. The standard of care consists of surgery and high-dose radiation therapy, better if with particle, due to their radioresistance and proximity to organs at risk such as brainstem and optic pathways. Due to the rarity of the tumor and its site, outcomes in terms of local control and toxicity of patients with this malignancy after receiving particle therapy has been documented only in a limited number of series with a restricted number of patients, in particular with regard to carbon ions. The aim of our retrospective study is to assess the role of particle therapy (protons and carbon ions) after surgery in our Institute in skull-base chondrosarcomas.

**Abstract:**

Background: The standard treatment for skull base chondrosarcoma (SB-CHS) consists of surgery and high-dose radiation therapy. Our aim was to evaluate outcome in terms of local control (LC) and toxicity of proton therapy (PT) and carbon ion (CIRT) after surgery. Materials and methods: From September 2011 to July 2020, 48 patients underwent particle therapy (67% PT, 33% CIRT) for SB-CHS. PT and CIRT total dose was 70 GyRBE (relative biological effectiveness) in 35 fractions and 70.4 GyRBE in 16 fractions, respectively. Toxicity was assessed using the Common Terminology Criteria for Adverse Events (CTCAE v5). Results: After a median follow-up time of 38 months, one local failure (2%) was documented and the patient died for progressive disease. Overall, 3-year LC was 98%. One (2%) and 4 (8%) patients experienced G3 acute and late toxicity, respectively. White-matter brain changes were documented in 22 (46%) patients, but only 7 needed steroids (G2). No patients had G3 brain toxicity. No G4–5 complications were reported. We did not find any correlation between high-grade toxicity or white-matter changes and characteristics of patients, disease and surgery. Conclusions: PT and CIRT appeared to be effective and safe treatments for patients with SB-CHS, resulting in high LC rates and an acceptable toxicity profile.

## 1. Introduction

Skull base chondrosarcoma (SB-CHS) is a rare malignant bone tumor arising from the chondrocytes or their precursor cells involved in the endochondral ossification, commonly at the petroclival junction, representing 6% of all skull base tumors and approximately 0.15% of all intracranial neoplasms [1,2].

CHS are subclassified into the conventional (most frequent), dedifferentiated, clear cell and mesenchymal subtypes. Conventional CHS can develop de novo or in a pre-existing enchondroma (in Ollier disease or Maffucci syndrome; secondary central tumors) and osteochondroma (secondary peripheral tumors) [3] and are classified according to World Health Organization’s (WHO) three histological classes: grade I (well differentiated), grade II (moderately differentiated) and grade III (poorly differentiated) [4]. Grading is important in CHS for prognosis and it is primarily based on nuclear size and staining, cellularity and number of mitoses. Grade I CHS are moderately cellular and contain hyperchromatic, plump nuclei of uniform size, without mitoses. Grade II CHSs are more cellular and cointain a greater degree of nuclear atypia, hyperchromasia and nuclear size; some mitoses can be found. In Grade III CHSs, mitoses are easily detected and lesions are more cellular and pleomorphic [5]. CHSs stain positive for S-100 and vimentin but fail to express epithelial markers such as cytokeratin and epithelial membrane antigen [6]. Low grade (WHO I) SB-CHSs rarely metastasize; in contrast, high grade CHSs, which make up to 5%–10% of all conventional CHSs are very aggressive and can metastasize to the lung [7].

As a slow-growing tumor that gradually progresses from abutting or encasing to subsequently invading critical organs, CHSs are often diagnosed at the development of symptoms caused by the presence of larger tumors [8].

Because of its deep location, with proximity to cranial nerves and major vascular structures, and its infiltrative growth pattern, gross-total surgical resections are achieved only rarely [9]. Despite the possibly long period of stability following subtotal resection, the majority of patients will experience recurrence after surgery alone [2,10]. Consequently, a combination of surgery, understood as maximum safe resection, and irradiation has become the mainstay of treatment either for naïve and recurrent patients, showing improvement in local control (LC) and overall survival (OS) [2]. CHSs are considered radioresistant cancers, although the mechanisms underlying the radioresistance have not been fully elucidated, including the ability to inhibit reactive oxygen species and the high expression of anti-apoptotic genes [11,12]. Thus, CHS respond best to high radiation doses over 70 Gy which cannot usually be reached by conventional radiotherapy (RT), due to the close proximity to dose-limiting critical structures (such as brainstem and optic pathways) [10,13,14,15].

Particle therapy, using protons and carbon ions, has the physical advantage of a finite range of dose deposition in brain tissue in depth with a steep dose fall-off after the Bragg peak, facilitating better normal tissue sparing, with a lower risk of radiation associated toxicities for a given dose [16]. Moreover, carbon ions have similar characteristics to protons, allowing high doses to be conformal to the target, with the further possible benefit of a higher relative biologic effectiveness compared to other modalities with respect to tumor cell death [17]. In fact, carbon ions can induce clustered DNA damage, that is difficult to repair and not subject to cell-cycle and oxygenation, whereas the simple DNA double-strand breaks produced by conventional RT are simpler to repair [17]. Consequently, carbon ions theoretically offer advantages due to enhanced biologic effectiveness in slow-growing and radioresistant tumors.

The purpose of this study was to evaluate results in terms of LC and toxicity of proton (PT) or carbon ion therapy (CIRT) of patients with SB-CHSs at the National Center for Oncological Hadrontherapy (CNAO).

## 2. Materials and Methods

### 2.1. Patient Selection

We reviewed the Institutional patient registry, collecting data of patients with histological diagnosis of SB-CHS treated with particle therapy at CNAO between September 2011 and July 2020. Inclusion criteria were: good performance status (Karnofsky Performance status ≥60), age ≥16 years old, follow-up of at least 6 months and particle therapy with curative intent. Exclusion criteria were: metastatic disease, no histological diagnosis, previous RT, concomitant chemotherapy and extensive metal instrumentation/implants. The local ethics committee approved the study (CNAO-OSS-21-2020) and written informed consent for treatment and the use of anonymized data for research and educational purposes was obtained from all participants and their legal guardians.

### 2.2. Treatment Planning and Delivery

All patients were treated in supine position. Immobilization was performed using customized thermoplastic head-masks and mouth-bites. A simulation computed tomography (CT) scan without contrast enhancement was acquired for treatment plan optimization and magnetic resonance imaging (MRI) sequences were performed with the same set-up conditions of the simulation CT. Subsequently, an image fusion between the CT and the diagnostic MRI was performed using non-deformable algorithms based on anatomical landmarks to delineate the target volumes and the organs at risk (OARs).

Gross tumor volume (GTV) included the visible tumor or area deemed to contain the tumor and it was manually delineated. T2 weighted axial images were routinely employed and contours were checked on post-contrast T1 images. High-risk clinical target volume (CTV) for possible microscopic disease extension was delineated around the GTV with 3–5 mm safety margins, modified according to both the anatomy and surgical pathway, to include the high-risk areas of tumor recurrence. Low-risk CTV included high-risk CTV and surgical resection margins around the pre-operative GTV modified according to both the anatomy and surgical pathway.

All patients were treated according to a sequential boost protocol. PT was administered once a day, 5 days per week, with a total prescription dose of 70 GyRBE (relative biological effectiveness)—Biological effective dose (BED) 116 Gy—delivered in 35 fractions of 2 GyRBE: 50 GyRBE to the low-risk volume and additional 20 GyRBE to the high-risk volume. CIRT prescription dose was 70.4 GyRBE—BED 173 Gy—delivered in 16 fractions of 4.4 GyRBE, 4 days per week: 39.6 GyRBE and 30.8 GyRBE to low-risk CTV and high-risk CTV, respectively. Biological effective dose (BED) was calculated both for PT and CIRT according to the formula:

BED = Total dose × [1 + (Fraction dose/*α/β*)] (1)

as in Kawashiro et al. [18], assumimg that CHSs are low *α/β* tissues [19].

Dose constraints for Organs at Risks (OARs) followed published data and clinical experience reported from other PT and CIRT series. Dose constraints to the OARs for PT were maximum dose (Dmax) of 54 and 63 GyRBE to the center and surface of the brainstem, respectively [20,21] and 60 GyRBE to the optic pathways [22]. Dose constraints to the OARs for CIRT were maximum dose of 30 GyRBE to the surface of the brainstem [23] until 2018, then we started to use D0.7 cm^3^ < 38 GyRBE and D0.1 cm^3^ < 46 GyRBE [24]. For optic pathways we considered Dmax < 40 GyRBE [23,25]. Dose contraints to temporal lobe were D2 cm^3^ < 70 GyRBE for PT [26] and D5 cm^3^ < 54 GyRBE [27].

A constant RBE value of 1.1 was applied for PT RBE-weighted dose calculation. We used the Local Effect Model I (LEM) to obtain RBE-weighted doses in CIRT treatment. We used a beam-specific virtual target margin of 2 mm in the lateral, proximal and distal direction for CIRT, 3 mm (lateral) and 2 mm (proximal and distal) for PT. In addition, when robust optimization was available (from 2019), we optimized all plans with a 2 mm patient position uncertainty and 3% range uncertainty.

The particle choice (proton or carbon ion) was not randomized, but personalized for each patient upon histological characteristics, toxicity patient-related risks and GTV volume at the baseline MRI. In the absence of macroscopic disease (GTV not identified), CIRT is usually not indicated in the clinical practice, so we had to choose PT in case of macroscopically complete resection. On the contrary, we usually chose CIRT especially in more aggressive disease: in case of high-grade CHSs, larger GTV and high proliferative index.

### 2.3. Follow-Up Evaluation

During treatment, each patient was examined by the dedicated radiation oncologist at the beginning of the treatment, then once a week. Toxicity data were collected in the personal patient chart. After the end of the treatment, each patient was monitored and followed up according to our institutional policy; acute and late effects were recorded at each follow-up examination. To evaluate tumor response, brain and head and neck MRI or CT were performed every 3–4 months after the end of the treatment for the first two years, every 6 months for the following three years, and then annually. Patients performed blood hormone assays, audiometric and visual examinations at the baseline then every year. Thorax CT or other imaging studies were ordered based on any evidence of metastasis. Acute (within 3 months of treatment) and late (more than 3 months after treatment) toxicities were classified according to the Common Terminology Criteria for Adverse Events (CTCAE) v5 grading (G) system [28].

### 2.4. Endpoints and Statistical Analysis

This is a retrospective monocentric study with the primary objective to evaluate the effectiveness in terms of LC in SB-CHS. Secondary endpoints of the study were toxicity profile and identification of prognostic factors on LC and toxicity. LC was defined as absence of progression by radiological assessment. Any enlargement of the lesion on subsequent radiological studies was considered a local recurrence.

Patient’s clinical-pathological and tumor characteristics were expressed as absolute and relative frequencies when variables are categorical or as median and interquartile range when presented as continuous variables. We used Fisher exact test to investigate differences in frequencies by toxicity and type of RT treatment. All analyses were carried out with SAS and two-sided *p*-values < 0.05 were considered statistically significant.

## 3. Results

Forty-eight patients with histological diagnosis of SB-CHS treated with PT or CIRT at CNAO between September 2011 and July 2020 were included in the current analyses. The characteristics of patients are detailed in Table 1.

The median age was 50 years (range, 17–77), 60% were female and 40% were male. A total of 37 patients (77%) had been referred to our Institute at the initial diagnosis (primary disease) and 11 patients (23%) at recurrence (recurrent disease). Gross total resection was performed in 3 patients (6%), incomplete macroscopic surgery in 36 patients (75%) and biopsy in 9 patients (19%). Median GTV in patients without gross total resection was 14 cm^3^ (range, 1.5–54.7). Of the 48 patients, 19 (40%) had a diagnosis of grade I CHS, 27 (56%) and 2 (4%) a diagnosis of grade II and grade III CHS, respectively.

Thirty-two (67%) patients received PT and the remaining 16 patients (33%) received CIRT.

The median follow-up for the entire group was 35 months (range, 7–93). The median follow-up times for the PT and CIRT group were 31 and 66.5 months, respectively.

The PT and CIRT subgroup did not differ for any characteristics such as age, sex, GTV volume and type of surgery (complete/incomplete/biopsy), however most of the patients with grade II and III SB-CHSs were treated with CIRT or PT (75% vs. 53%).

### 3.1. Local Control

At the time of analysis, tumors in 47 patients (98%) remained locally controlled and in only one patient (2%) local recurrence had been identified. Local recurrence occurred after 21 months from the end of the treatment and resulted in the death of the patient a few months after recurrence. According to actuarial analysis, our data resulted in a 3-year LC rate of 98% in all the study population, while 3-year LC rates for PT and CIRT subgroup were 100% and 94%, respectively. Thus far, no patient has developed distant metastasis.

Treatment failure occurred in a 76-year old male patient, with grade I SB-CHS who underwent only biopsy before CIRT because of the deep position of the disease. GTV was 7.35 cm^3^ and it was localized in close proximity to the brainstem. Consequently, dose levels delivered to 95% of GTV volume (D95%), D99 and Dmean were 35.9, 23.6 and 63 GyRBE, respectively.

As there was only one local relapse, no further analysis was possible on possible prognostic factors on LC (age, sex, the presence/absence of comorbidities such as diabetes and hypertension, outcome and number of surgeries, surgical technique, brainstem and optic pathway involvement and GTV volume).

### 3.2. Acute Toxicity

Most of the patients had varying degrees and combinations of acute G1–2 toxicities during PT or CIRT, such as fatigue, radiation dermatitis, mucositis, alopecia and middle ear effusion. All side effects were controlled symptomatically. A total of 21 (41%) patients developed G1–2 skin or mucosal toxicity during treatment. Only one patient (2%) developed G3 acute mucositis, but no discontinuation of RT or hospitalization was necessary and the patient had a complete resolution of symptoms within 3 months after the end of treatment. No G4–G5 acute toxicity occurred.

### 3.3. Late Toxicity

There have been 4 (8%) grade G3 late toxicity to date after a median time of 53 months (Table 2). Two patients with late G3 toxicity received PT; the remaining 2 received CIRT (*p* = 0.59). No G4–5 late complications were reported.

One patient required hospitalization after 70 months from PT for the occurrence of severe hyperosmolar hyponatremia in central G3 hypoadrenalism. Afterwards, he was diagnosed with hypogonadism and hyperprolactinemia and he had to start hormone replacement therapy.

In another patient G3 toxicity (hearing loss) was reported after PT. After 17 months from the end of the treatment, she performed a pure tone audiometry with the finding of severe hearing-impairment requiring aids. During PT and in the following weeks, she experienced G2 otitis and inflammation of the mastoid air cells.

One patient treated with CIRT experienced recurrent acute and late middle ear effusion, which was often treated with antibiotics until she required intervention (myringotomy with positioning of a tympanostomy tube) for G3 otitis after 50 months from the end of the treatment.

A G3 osteonecrosis of clivus was reported in a woman after 57 months from the end of CIRT. She presented with pain, foul odor and had exposed and necrotic bone. She received antibiotics for concomitant polymicrobial infection and then she underwent hyperbaric oxygen therapy without effect. Consequently, a surgical debridement approach of the necrotic and devascularized bone was performed via endonasal endoscopic approach.

**Figure 1:**
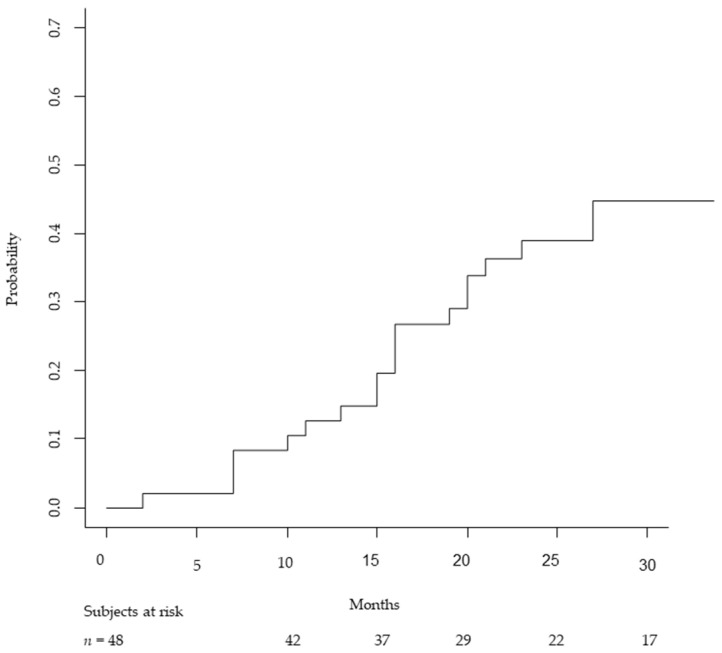
Cumulative incidence showing the cumulative brain injury (G1 and G2) rates over time in PT and CIRT.

In addition to high-grade toxicity, we analyzed the occurrence of white-matter changes in the temporal lobe (Table 2). These radiological alterations in temporal lobe tissue (brain injury) were found in 22 patients (46%): 15 were asymptomatic (G1 toxicity) while the remaining 7 suffered from symptoms including headaches, occasional dizziness, impairment of memory and personality changes and seizures, so they had to start medical treatment such as steroids (G2 toxicity). No hospitalization was needed and symptoms were under control after starting medical therapy. These temporal lobe white-matter changes occurred after a median time of 16 months after particle therapy and were found in 40% of patients treated with PT and 56% of patients receiving CIRT, without a statistically significant difference between the two subgroups (*p* = 0.36). In Figure 1 we report the cumulative incidence of brain injury (symptomatic and asymptomatic) in our series. Median time to the occurrence of lobe white-matter changes was 16 months (range, 2–40).

We evaluated the possible relationship between toxicity events (late G3 and brain injury of any grade) and potential risk factors. Specifically, we investigated sex, age, comorbidities such as diabetes and hypertension, number of surgeries, type of surgery and GTV volume, but none of these risk factors were statistically predictive of G3 late toxicity or the occurrence of a brain injury of any grade.

## 4. Discussion

The results of the present study, despite the limited number of patients due to the rareness of CHS, showed that high dose PT and CIRT achieved high LC for SB-CHS with acceptable radiation-induced toxicity.

Our results on LC are in line with data from previous studies, which are mostly larger retrospective series of skull base neoplasms including both chordomas and CHSs. As demonstrated in Table 3, in SB-CHS the 3 to 5 year LC ranges from approximately 86% to 100% [13,14,29,30,31,32,33]. Chordomas, which are often combined with CHSs in retrospective analyses, however, have slightly lower LC and survival rates than CHSs [14,34,35]. Although radiation dose and volumes for skull base chordoma and SB-CHS are similar, we focused our analysis only on CHS due to the difference in terms of histologic and immunohistologic features, as well as different natural history and clinical outcomes between chordoma and CHS.

In our series, we did not report relapses in the subgroup of SB-CHS treated with PT. A possible explanation could be the shorter follow-up time in PT (31 months) than in CIRT patients (66.5 months). With a time of 21 months between therapy and progression in our studies but a median of 23.4–29.8 months in other trials [14,32], relapses in the PT subgroup could have not been detected yet due to the shorter follow-up interval for many patients. Indeed, patients had a follow-up time of less than 21 months after PT. The difference between PT and CIRT in follow-up duration could be attributed to the fact that most carbon ion treatments were performed between 2013 and 2015, while PT has been more frequently prescribed in later years. For this reason, the most recently treated patients have not yet achieved an adequately long follow-up like the first patients treated with CIRT.

We did not observe a relationship between recurrence and patient’s and tumor’s characteristics, which may be attributable to the small sample size and the low rate of failure.

Previous studies reported optic apparatus and/or brainstem compression as a major prognostic factor for patient’s outcome [13,14,34]. When a tumor abuts a critical structure, GTV will not receive the prescribed radiation dose to its entire volume and, hence, it is likely to be insufficient for tumor control. In our study, the patient who experienced local recurrence had the lesion in close proximity with the brainstem, without a satisfactory dose coverage to GTV. Consequently, local recurrence occurred precisely deeply towards the brainstem. The relapse showed a very fast growth and the patient died a few months later without being able, due to the location of the disease and his comorbidities, to receive a salvage surgery. Therefore, it is not possible to know the histological characteristics of the lesion, in instances of a suspicion of a dedifferentiation or a greater aggressiveness of the disease. Because the chances of new treatments for recurrence are often remote as in our series, the possibility to deliver an adequate radiation dose to SB-CHSs is essential to improve LC.

Moreover, the patient with relapse of disease was treated in 2013, a year after CNAO started its treatment activity. In the first years of activity, the dose constraint to the brainstem at CNAO was therefore set to be D1% < 30 GyRBE, following the tradition of the National Institute of Radiological Sciences (NIRS, Chiba, Japan) [36], using hypofractionated treatment schedules. Afterwards, the original brainstem dose constraint applied at CNAO was proved to be too conservative compared with the clinical practice in Japan and new constraints, D0.7 cm^3^ < 38 GyRBE and D0.1 cm^3^ < 46 GyRBE, have been introduced in the prospective treatment protocols since October 2018 [23].

Our study shows the effectiveness of protons and carbon ions in the treatment of SB-CHSs, however PT and CIRT subgroups cannot be compared. The choice of the particle does not come from a random allocation, but rather it is personalized to each patient considering clinical and radiological characteristics, e.g., in case of gross total resection (GTV not identified), CIRT is usually not indicated in the clinical practice and we prefer to prescribe CIRT in case of more aggressive histology (WHO grade II and III). Not surprisingly, there is a statistically significant difference between the two groups regarding the histology of CHS.

Concerning safety, only a few severe treatment–related complications (8%) have been observed to date, without any life-threatening or fatal events. High grade G3 toxicity data do not differ significantly from what is reported in the literature, from 4% to 8% in skull base series mentioned above [13,14,33].

On the other side, the rate of occurrence of radio-induced changes in brain tissue and in temporal lobes in particular (46%) seems to be higher in our series than in previous literature.

In our opinion, these results should be interpreted with caution, especially due to the frequency of brain MRI performed during a patient’s follow-up that helps to detect asymptomatic brain changes that would otherwise be subclinical. This should be taken into consideration, especially when compared with older studies, when MRI was not always a routine exam in follow-up and when not all recent and advanced techniques, including diffusion, perfusion imaging, and spectroscopy, were performed. Moreover, in our series, we reported every radiological change in white matter, whether they manifested with edema, with contrast enhancement or with areas of necrosis [37], regardless of the clinical symptoms. In the comparison of the radio-induced brain changes’ rates among different series, the criteria adopted to estimate, to rate and to term this outcome can be rather heterogeneous.

The presence of clinical symptoms (CTCAE ≥2) associated with radiological changes and clinical/radiological improvement rates (spontaneous or induced by medical therapy as steroids or bevacizumab) could be considered more reliable criteria to compare different series. Besides that, no severe G ≥3 radiation necrosis was reported, as in previous trials [14,33,38].

We are aware that several limitations can be highlighted. Firstly, it is a monocentric retrospective analysis. Second, because of the small number of patients, the impact of the statistical analyses is relatively low. Third, CHS is a disease that takes a long time before local relapse and the follow-up period in this study, especially for the PT subgroup, is not sufficient. Moreover, given the significant diversity between the low-and high-grade CHSs, whether therapeutic strategies could be adapted according to differentiation grade and identification of biomarkers is largely unknown. However, all of the studies in literature on SB-CHSs are retrospective and including any WHO grade and performing a prospective/randomized study is difficult in cases of rare tumors.

## 5. Conclusions

We report a series of patients with SB-CHS patients who underwent skull base surgery followed by particle therapy. High dose particle therapy such as PT or CIRT following an appropriate surgical resection achieved local tumor control in the majority of patients.

Considering the high doses delivered, toxicity rates observed have been acceptable, given the few severe complications and the absence of life-threatening or fatal events.

## Figures and Tables

**Table 1:** Characteristics of patients.

Characteristics	Value	Number of Patients *n* = 48	%
Sex	F	29	60
	M	19	40
Genetic syndrome	no	44	92
	yes	4	8
Hypertension	no	34	71
	yes	14	29
Diabetes	no	44	92
	yes	4	8
Histology grade according WHO	G1	19	40
	G2	27	56
	G3	2	4
Outcome of surgery	gross total resection	3	6
	incomplete surgery	36	75
	biopsy	9	19
Number or surgeries	1	34	71
	2	9	19
	3	4	8
	4	1	2
Surgical technique	TNS	28	58
	craniotomy	15	31
	combined	5	11
Brainstem involvement	no	44	92
	yes (abutment)	1	2
	yes (compression)	3	6
Optic pathways involvement	no	40	83
	yes (abutment)	6	13
	yes (compression)	2	4
Timing of treatment	first diagnosis	37	77
	at the relapse	11	23
Treatment intent	post-operative	41	85
	primary	7	15
Particle	CIRT	16	33
	PT	32	67

F: female, M: male, WHO: World Health Organization, G: grade, TNS: Transnasal surgery, CIRT: carbon ion therapy, PT: proton therapy.

**Table 2:** Late toxicity and brain injury.

Toxicity	CIRT *n* = 16 (%)	PT = 32 (%)	Total = 48 (%)	*p*-Value *
Late toxicity				
G0–G2	14 (87.5)	30 (93.8)	44 (91.7)	0.59
G3	2 (12.5)	2 (6.3)	4 (8.3)	
Brain injury				
G0	7 (43.8)	19 (59.4)	26 (54.2)	
G1–G2	9 (56.3)	13 (40.6)	22 (45.8)	0.36

G: grade, CIRT: carbon ion therapy, PT: proton therapy, * Fisher Exact test.

**Table 3:** Single institutional reports on PT and CIRT for chondrosarcoma of the skull base.

Study, Year	Particle	Number of Patients	Prescription Dose (GyRBE)	Median Time of Follow-Up (Months)	LC Rate	Late Toxicity
Hug et al., 1999 [13]	Protons	25	70.2 * (median)	33.2 *	3 y LC: 94%	7% (G3–G4)
Munzenrider et al., 1999 [29]	Protons	229	72 * (mean)	41 *	5 y LC: 98%	-
Ares et al., 2009 [30]	Protons	22	68.4 (median)	34 *	5 y LC: 94%	6.2%
Fuji et al., 2011 [31]	Protons	8	63 * (median)	42 *	3 y LC: 86%	No G ≥3
Weber et al., 2016 [14]	Protons	71	72.5 * (median)	50 *	5 y LC: 93.6%	8.1% (G3–G4)
Mattke et al., 2018 [32]	Protons	22	70 (median)	30.7	4 y LC: 100%	No G ≥3
	Carbon ions	79	60 (median)	43.7	4 y LC: 90.5%	
Holtzman et al., 2019 [33]	Protons	43	73.8 (median)	44	4 y LC: 89%	4.6% (G3) + 9% G3 expected hear loss
Present study, 2021	Protons	32	70	31	3 y LC: 100%	8% (G3)
	Carbon ions	16	70.4	66	3 y LC: 94%	No G4–G5

LC: local control, G: grade, y: years, GyRBE: relative biological effectiveness,* data coming from mixed series on SB-CHS and chordoma.

## Data Availability

Data available on request due to privacy restrictions.
